# Clinically Relevant Concentrations of Polymyxin B and Meropenem Synergistically Kill Multidrug-Resistant *Pseudomonas aeruginosa* and Minimize Biofilm Formation

**DOI:** 10.3390/antibiotics10040405

**Published:** 2021-04-08

**Authors:** Hasini Wickremasinghe, Heidi H. Yu, Mohammad A. K. Azad, Jinxin Zhao, Phillip J. Bergen, Tony Velkov, Qi Tony Zhou, Yan Zhu, Jian Li

**Affiliations:** 1 Infection and Immunity Program, Department of Microbiology, Biomedicine Discovery Institute, Monash University, Clayton, VIC 3800, Australia; heidi.yu@monash.edu (H.H.Y.); mohammad.azad@monash.edu (M.A.K.A.); jinxin.zhao@monash.edu (J.Z.); phillip.bergen@monash.edu (P.J.B.); yan.zhu@monash.edu (Y.Z.); jian.li@monash.edu (J.L.); 2 Department of Pharmacology and Therapeutics, School of Biomedical Sciences, Faculty of Medicine, Dentistry and Health Sciences, The University of Melbourne, Parkville, VIC 3053, Australia; tony.velkov@unimelb.edu.au; 3 Department of Industrial and Physical Pharmacy, Purdue University, West Lafayette, IN 1047907, USA; tonyzhou@purdue.edu

**Keywords:** polymyxin, meropenem, combination therapy, multidrug resistance, *Pseudomonas aeruginosa*, synergy, biofilm

## Abstract

The emergence of antibiotic resistance has severely impaired the treatment of chronic respiratory infections caused by multidrug-resistant (MDR) *Pseudomonas aeruginosa*. Since the reintroduction of polymyxins as a last-line therapy against MDR Gram-negative bacteria, resistance to its monotherapy and recurrent infections continue to be reported and synergistic antibiotic combinations have been investigated. In this study, comprehensive in vitro microbiological evaluations including synergy panel screening, population analysis profiling, time-kill kinetics, anti-biofilm formation and membrane damage analysis studies were conducted to evaluate the combination of polymyxin B and meropenem against biofilm-producing, polymyxin-resistant MDR *P. aeruginosa*. Two phylogenetically unrelated MDR *P. aeruginosa* strains, FADDI-PA060 (MIC of polymyxin B [MIC_polymyxin B_], 64 mg/L; MIC_meropenem_, 64 mg/L) and FADDI-PA107 (MIC_polymyxin B_, 32 mg/L; MIC_meropenem_, 4 mg/L) were investigated. Genome sequencing identified 57 (FADDI-PA060) and 50 (FADDI-PA107) genes predicted to confer resistance to a variety of antimicrobials, as well as multiple virulence factors in each strain. The presence of resistance genes to a particular antibiotic class generally aligned with MIC results. For both strains, all monotherapies of polymyxin B failed with substantial regrowth and biofilm formation. The combination of polymyxin B (16 mg/L)/meropenem (16 mg/L) was most effective, enhancing initial bacterial killing of FADDI-PA060 by ~3 log_10_ CFU/mL, followed by a prolonged inhibition of regrowth for up to 24 h with a significant reduction in biofilm formation (* *p* < 0.05). Membrane integrity studies revealed a substantial increase in membrane depolarization and membrane permeability in the surviving cells. Against FADDI-PA107, planktonic and biofilm bacteria were completely eradicated. In summary, the combination of polymyxin B and meropenem demonstrated synergistic bacterial killing while reinstating the efficacy of two previously ineffective antibiotics against difficult-to-treat polymyxin-resistant MDR *P. aeruginosa*.

## 1. Introduction

Infections caused by multidrug-resistant (MDR) *Pseudomonas aeruginosa* present a serious challenge in clinical practice. This particularly troublesome opportunistic pathogen is notoriously difficult to treat, given its intrinsic resistance to many antibiotics and high propensity to develop resistance to all currently available antipseudomonals with monotherapy [1]. In the United States alone, over 32,000 cases of MDR *P. aeruginosa* were detected in 2017, resulting in 2700 deaths [2]. Indeed, the World Health Organization (WHO) designated carbapenem-resistant *P. aeruginosa* a Priority 1 (Critical) organism urgently requiring novel treatment options [3]. Unfortunately, the drug development pipeline is struggling to match the demand for new antibiotics, including those active against *P. aeruginosa* [4,5]. Consequently, alternative treatment strategies to combat this MDR pathogen, including the improved use of currently available antibiotics, are urgently required.

The emergence of MDR Gram-negative organisms to more recently introduced antibiotics has forced clinicians to re-examine ‘old’ antibiotics such as the polymyxins [6]. The polymyxins are a multi-component class of non-ribosomal, poly-cationic lipopeptides, and only polymyxin B and E (colistin) are clinically available [7]. Polymyxin B and colistin differ by a single amino acid and have virtually identical potencies and spectrums of activity [7]. This class of antibiotics was once abandoned due to significant toxicity concerns, primarily nephrotoxicity which may occur in up to 60% of patients receiving intravenous polymyxin therapy [8]. However, given the polymyxins retain significant in vitro activity against many Gram-negative MDR bacteria, including *P.* *aeruginosa* [9,10], their use has steadily increased over the last two decades. In many cases, they are the only therapeutic option available to treat infections caused by these MDR pathogens [11].

As is inevitable with any antibiotic, increased polymyxin use has resulted in the emergence of polymyxin resistance in patients, which is beginning to spread worldwide, threatening the utility of this increasingly important last-line therapeutic option [12]. The potential for the rapid emergence of polymyxin resistance even with supra-clinical concentrations has been well demonstrated in pre-clinical studies, including against *P. aeruginosa* [13]. To combat this, polymyxin combination therapy has been proposed as one way to increase antimicrobial activity and reduce the development of resistance [14]. Consequently, polymyxin-based combinations are increasingly used clinically to treat infections caused by MDR Gram-negative organisms, despite a lack of evidence-based treatment guidelines. In such combinations, polymyxins are administered either intravenously and/or, for lung infections, via inhalation [15,16]. Therefore, the current study sought to identify an effective, clinically translatable polymyxin B-based combination therapy against biofilm-producing, polymyxin-resistant MDR *P. aeruginosa*. Clinically relevant concentrations of each drug alone and in combination were used to examine bacterial killing of planktonic bacteria, inhibition of biofilm formation, as well as sub-population modulations of the proposed combination via membrane integrity studies.

## 2. Results

### 2.1. Genome Sequencing, Resistance Genes and Virulence Factors

Genome sequencing yielded approximately 7.6 million reads per sample, equivalent to 2.3 Gb data per isolate. *De novo* assembly generated 47 and 87 contigs (≥1000 bp) for FADDI-PA060 and FADDI-PA107, respectively, with the corresponding total length of 6,371,158 and 6,266,040 bp; N50 of 371,572 and 419,976 bp; and GC content of 56.9–57.3%. In total, 5924 and 5787 genes were annotated with 5852 and 5709 coding proteins for FADDI-PA060 and FADDI-PA107, respectively.

**Figure 1:**
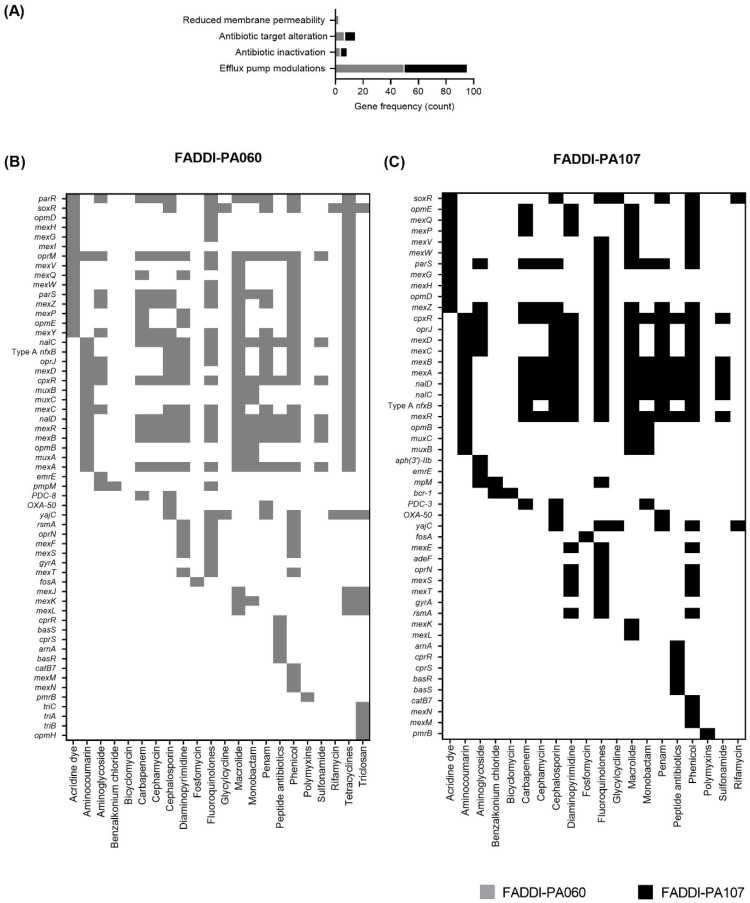
Genome-based antimicrobial resistance predictions for FADDI-PA060 and FADDI-PA107. Frequency of known antimicrobial resistance conferring functional products in FADDI-PA060 and FADDI-PA107 (**A**). Predicted antimicrobial resistance profiles of FADDI-PA060 (**B**) and FADDI-PA107 (**C**). The presence of a gene conferring resistance to a specific antibiotic class is marked by a shaded square.

**Figure 2:**
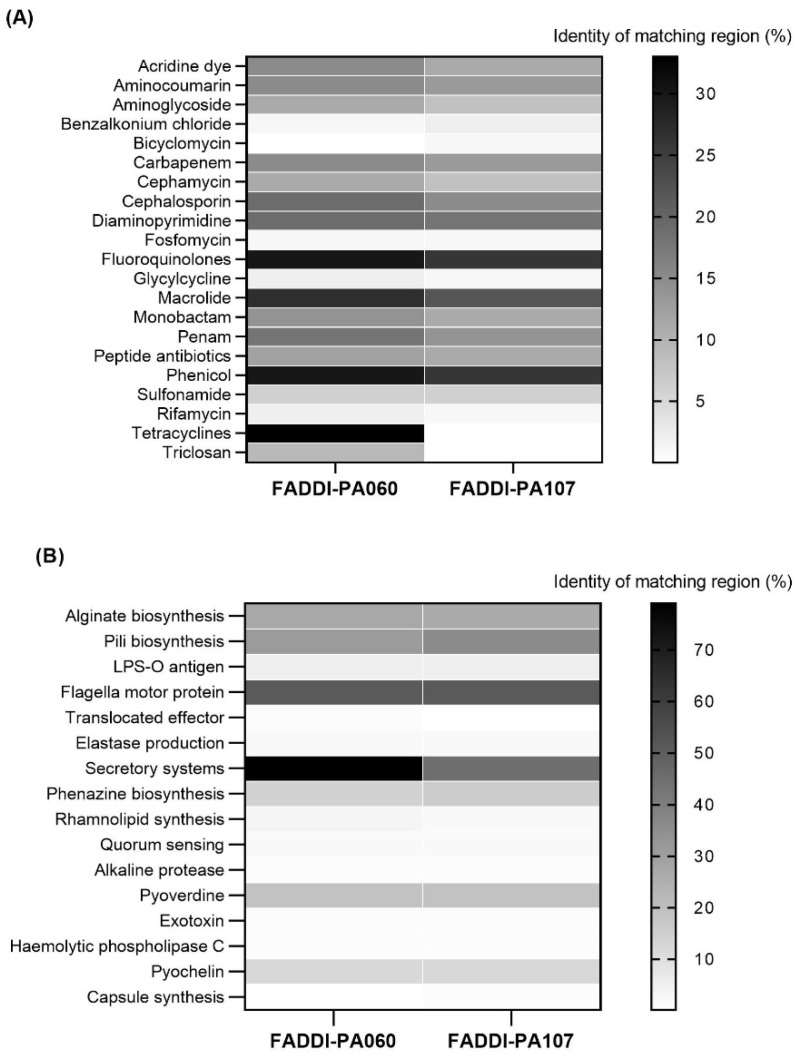
Abundance of genes predicted to confer resistance to different classes of antibiotics (**A**) and determinants of virulence factors contributing to prolonged pathogenesis (**B**). The scales to the right indicate the protein sequence identity (%) of the matching region for each determinant.

A total of 57 genes in FADDI-PA060 and 50 genes in FADDI-PA107 were predicted to confer resistance to a variety of antimicrobials (Figure 1 and Figure 2A). Of particular interest was the presence of 8 point mutations in each strain on genes encoding the PmrAB two-component regulatory system, a key system involved in the development of polymyxin resistance in *P. aeruginosa* [17]. A total of 15 genes in FADDI-PA060 and 13 genes in FADDI-PA107 are involved with carbapenem resistance, including *bla*_PDC-8_, *bla*_PDC-3_, *cpxR*, *mexAB*, *mexPQ*, *mexYZ*, *mexR, nalCD*, *nfxB, oprM*, *opmE* and *parRS* in both isolates. The presence of genes such as *mexD, oprJ, mexC, mexY, parS, mexZ, parR, oprM* and *cpxR,* previously reported to be associated with macrolide resistance indicated the likelihood of bacterial innate resistance to this class of antibiotics [18]. Other identified genes in each strain conferred resistance to many antibiotic classes, including cephalosporins and fluoroquinolones. Furthermore, *soxR* and *yojC*, which confer resistance to rifamycins, were identified in each strain.

In FADDI-PA060 and FADDI-PA107, 249 and 220 putative virulence factors were identified, respectively (Figure 2B). Both strains contained genes encoding for major virulence categories including adherence (alginate and pili biosynthesis), motility (motor, flagellin and chemotaxis proteins biosynthesis), regulatory system modulators, secretion system proteins and haem-regulatory proteins. Many of these genes are genus-specific and have been previously reported to enhance pathogenesis [19,20]. Of particular interest are the alginate genes present in both strains which are reported to play a role in the establishment and formation of the polymeric matrix of biofilms [21].

### 2.2. Susceptibility, Baseline Population Analysis Profiles (PAPs) and Checkerboard Assay

**Figure 3:**
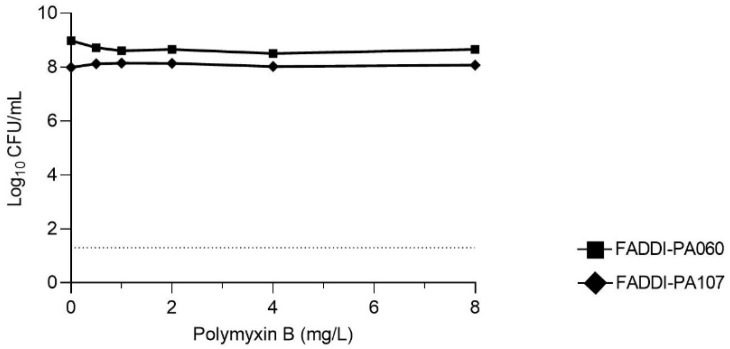
Baseline population analysis profiles of FADDI-PA060 and FADDI-PA107 at an initial inoculum of ~10^8^ CFU/mL. The limit of detection is shown by the dotted horizontal line at 1.30 log_10_ CFU/mL.

**Figure 4:**
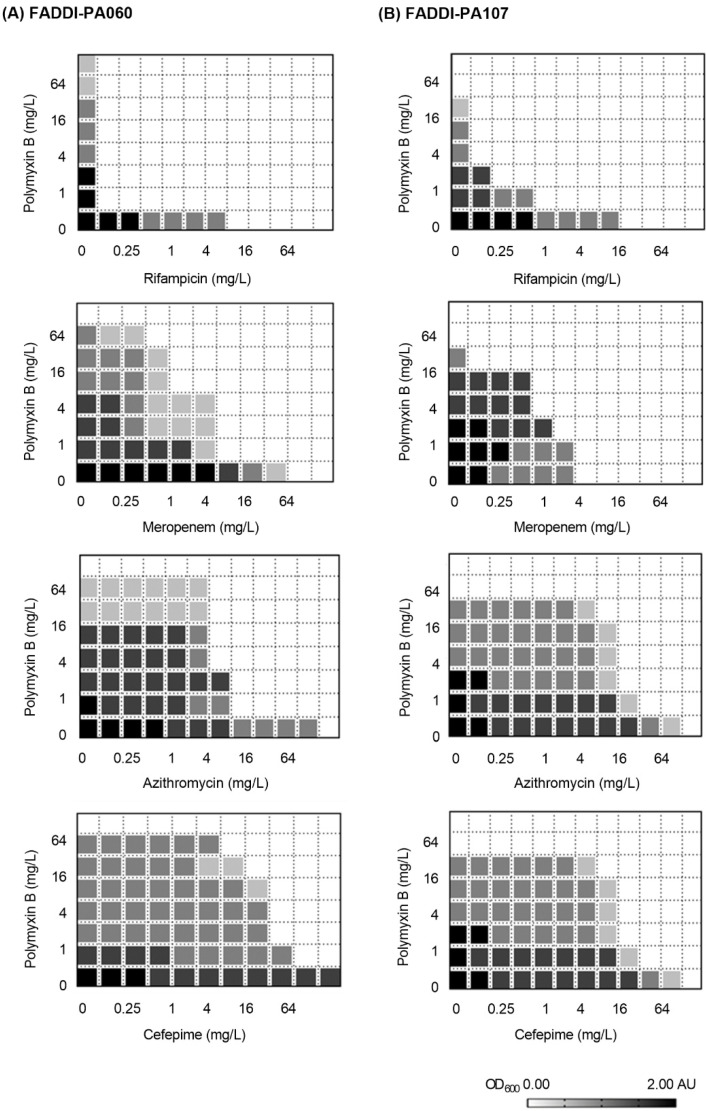
Checkerboard assay results of FADDI-PA060 (**A**) and FADDI-PA107 (**B**) examining polymyxin B in combination with a representative antibacterial agent from different antibacterial classes. Color gradient corresponds to the average turbidity at λ = 600 nm.

The MICs of both isolates to all antibiotics studied are shown in the Table 1. As suggested by the genetic antibiotic resistance profiles (Figure 1 and Figure 2), each isolate was MDR according to the criteria of Magiorakos et al. [22] and resistant to polymyxins. The PAPs prior to antibiotic exposure (i.e., at baseline) are shown in Figure 3. Consistent with the high polymyxin B MICs (Table 1), virtually the entire bacterial population of each isolate at this high inoculum (~10^8^ CFU/mL) grew in the presence of 16 mg/L polymyxin B, indicating the absence of baseline sub-population heterogeneity. Results for the checkerboard assay are shown in Figure 4. Synergy was most pronounced with polymyxin B in combination with meropenem and rifampicin. Therefore, the combination of polymyxin B and meropenem was chosen for further evaluation in time-kill and biofilm studies.

### 2.3. Anti-Biofilm Effects and Time-Kill Study

**Figure 5:**
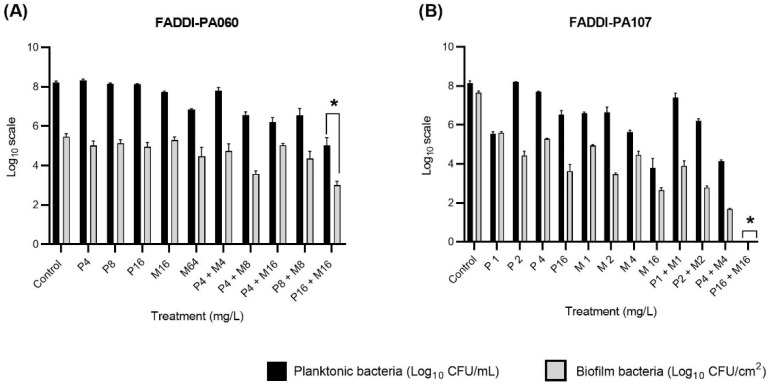
Planktonic and biofilm growth of MDR *P. aeruginosa*; FADDI-PA060 (**A**) and FADDI-PA107 (**B**) following 24 h exposure to polymyxin B (P) and/or meropenem (M) in the dynamic biofilm model. Planktonic cell counts are a measure of observed viable cell counts per mL whereas biofilm cell counts are a measure of observed viable cell counts per cm^2^ of the Teflon coupons (the aggregating surface for biofilm formation). One-way ANOVA with Kruskal–Wallis multiple comparisons versus control group * *p* < 0.05. Each experiment was performed in triplicate and data are presented as mean ± SD (*n* = 3).

Preliminary biofilm formation studies indicated substantial biofilm formation in the absence of antibiotic treatment following 24 h incubation (Figure S1), with no significant difference in biofilm formation following 24 or 48 h incubation (48 h data not shown). Biofilm formation was also reduced to varying degrees with each monotherapy and the combination (Figure S1). Based on these results, 24 h was selected as the preferred incubation period to analyze the initial stages of biofilm development in the dynamic model. Bacterial growth at 24 h in the dynamic biofilm-formation studies is shown in Figure 5. For FADDI-PA060, significant reductions in planktonic and biofilm growth (* *p* < 0.05) compared to the control and monotherapy groups were only observed following exposure to the polymyxin B/meropenem combination, each at 16 mg/L (Figure 5A). FADDI-PA107 responded similarly, with no detectable planktonic or biofilm-embedded bacteria at 24 h with the same combination therapy (Figure 5B). Similarly, growth of planktonic bacteria above 4 log_10_ CFU/mL and the establishment of a biofilm occurred with all other regimens against both isolates (Figure 5).

**Figure 6:**
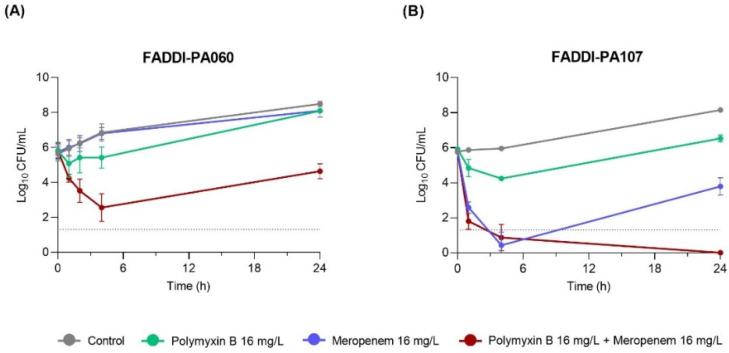
Time-kill curves of FADDI-PA060 (**A**) and FADDI-PA107 (**B**) with polymyxin B 16 mg/L and meropenem 16 mg/L, alone and in combination, at an inoculum of ~10^6^ CFU/mL. The limit of detection is shown by the dotted horizontal line at 1.30 log_10_ CFU/mL. Time-kill studies were performed in triplicate and data are presented as mean ± SD (*n* = 3).

Based on the anti-biofilm results described above, the combination of polymyxin B and meropenem (each at 16 mg/L) was further examined in a time-kill study (Figure 6). Both monotherapies were ineffective against the meropenem-resistant isolate FADDI-PA060 (meropenem MIC, 64 mg/L; Figure 6A). While regrowth was observed at 24 h, the combination had at least 3 log_10_ CFU/mL killing within 4 h. Against the meropenem-susceptible isolate FADDI-PA107 (meropenem MIC, 4 mg/L), polymyxin B alone was ineffective, but rapid and substantial bacterial killing (~5 log_10_ CFU/mL by 4 h) occurred with both meropenem monotherapy and the combination (containing meropenem at 4 × MIC; Figure 6B). At 24 h, substantial regrowth (to ~4 log_10_ CFU/mL) had occurred with meropenem monotherapy, while no viable bacteria were detected with the combination.

### 2.4. Membrane Integrity Assessment

**Figure 7:**
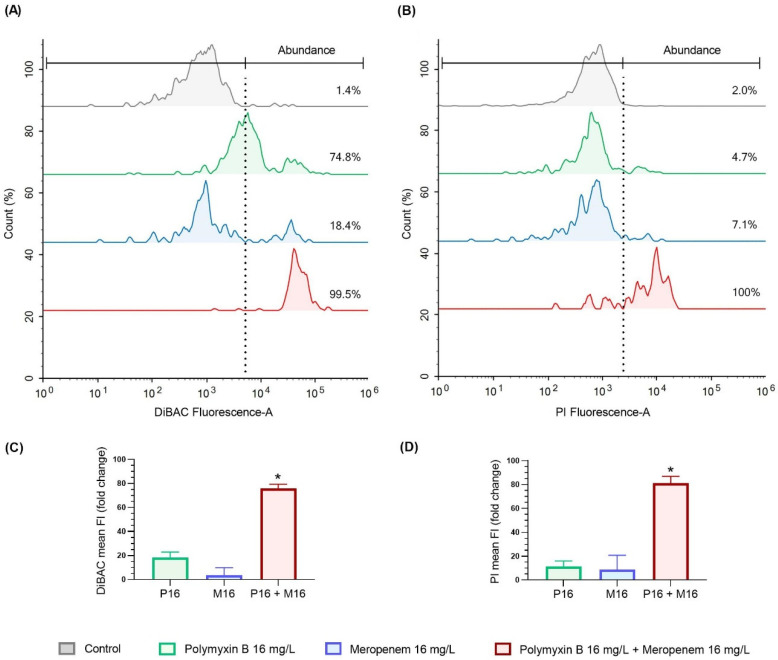
Membrane integrity of treatment-resistant MDR *P. aeruginosa* FADDI-PA060 at 4 h post-exposure to polymyxin B and/or meropenem (each at 16 mg/L). Treatment-driven subpopulation-based transference of DiBAC (**A**) and PI (**B**) fluorescence curves indicating the abundance of the population (%) with increased fluorescence intensity compared to the control group (segregated with a dotted vertical line). Changes in fluorescence intensity (FI) of DiBAC (**C**) and PI (**D**) are presented as the fold change in mean fluorescence intensity normalized to the non-treated control. One-way ANOVA comparison of treatment group vs. control group (* *p* < 0.05). Data are presented as mean ± SD (*n* = 3).

As the proposed combination of 16 mg/L of polymyxin B and meropenem resulted in bacterial eradication against FADDI-PA107 in the time kill studies (Figure 6B), not enough cells from this isolate were available for further analysis. Consequently, surviving FADDI-PA060 bacterial cells at 4 h post treatment, the time at which maximal bacterial killing was observed prior to regrowth (Figure 6A), were employed for membrane integrity studies. Treatment-induced changes to membrane polarity and permeability for FADDI-PA060 are shown in Figure 7.

Membrane depolarization as determined by an increase in DiBAC fluorescence intensity (FI) [24], indicated that following polymyxin B monotherapy, a substantial shift in the fluorescence curve occurred with 74.8% of the population having a FI greater than that of the control group (Figure 7A,C). Within this 74.8%, a smaller subpopulation of cells had substantially higher DiBAC FI, indicating greater membrane depolarization amongst these cells (Figure 7A). With meropenem monotherapy, 81% of the population had a membrane polarity similar to that of the control group, with the remaining 18.4% of the cells experiencing a high degree of depolarization similar to the depolarized subgroup observed with polymyxin B monotherapy (Figure 7A,C). However, following exposure to the combination therapy, 99.5% of the population experienced a high level of membrane depolarization similar to that of the subpopulations observed with each monotherapy (Figure 7A), with the mean FI significantly (* *p* < 0.05) higher than the control and monotherapy groups (Figure 7C). Membrane permeability as assessed by propidium iodide fluorescence [25], provided similar results to that of DiBAC. Namely, a small subpopulation of cells having increased membrane permeability with both monotherapies and, with combination therapy, movement of the entire fluorescence curve to the right with virtually all cells having significantly (* *p* < 0.05) high PI fluorescence and therefore, substantially increased membrane permeability (Figure 7B,D).

## 3. Discussion

The discovery of antibiotics has revolutionized modern medicine [26]; however, resistance seems inevitable with almost any antibiotic and identifying novel bacterial targets has proved challenging [27]. Indeed, the WHO recently identified only 32 antibiotics in clinical development that target high-priority multidrug-resistant organisms, with only six classified as innovative (https://www.who.int/news-room/fact-sheets/detail/antimicrobial-resistance, accessed on 7th April 2021). Thus, there is an urgent need to optimize currently available therapeutics [26]. Given the scarcity of new antibiotics with activity against MDR Gram-negative bacteria in the antibiotic discovery pipeline, polymyxins remain a critical last-line therapy against MDR Gram-negative pathogens [11]. However, with resistance to polymyxins increasing [12], polymyxin-based combinations are frequently used in an attempt to increase antimicrobial activity and reduce resistance development, despite a lack of evidence-based treatment guidelines. It is therefore crucial that such combinations are optimized. In this study, we employed two phylogenetically unrelated clinical strains of *P. aeruginosa*, FADDI-PA060 and FADDI-PA107. FADDI-PA060 is a closely related sequence type to the DK-2 clone types found in cystic fibrosis patients over the last 35 years [28], whereas the genomic analysis reported here identified FADDI-PA107 as a newly emerging, human-related sequence type. Genomic analysis permitted the prediction of the antibacterial resistance profiles as well as virulence of the strains of interest (Figure 1 and Figure 2), with both isolates harboring many antibiotic resistance genes (55 genes in FADDI-PA060 and 52 genes in FADDI-PA107). Genes included those encoding resistance to fluoroquinolones, macrolides, aminoglycosides, carbapenems and cephalosporins. Generally, the presence of resistance genes to a particular category of antibiotic aligned with resistance as determined by MICs, with some minor differences. Specifically, with isolate FADDI-PA107, the MICs to ciprofloxacin and meropenem were <0.125 mg/L and 4 mg/L (both considered susceptible), respectively, despite the presence of genes conferring resistance to both antibiotics. In both cases, MICs were retested with the original MICs confirmed.

Systematic evaluation of virulence prediction genes yielded a total of 249 genes in FADDI-PA060 and 220 genes in FADDI-PA107 (Figure 2B). These genetic determinants of virulence indicated that both strains were biofilm-producing, which was subsequently confirmed in vitro. The abundance of antibiotic resistance and virulence factor-determining genes in both isolates is in agreement with the resistance to common antibiotics and prolonged disease progression observed with the DK-2 clonal types mentioned above [28,29,30] and suggests that FADDI-PA107 would likely cause similar problems. Having determined the resistance profiles and virulence factors of the two isolates, we undertook preliminary screening of polymyxin-based antibiotic combinations using multiple clinically relevant concentrations to identify potentially efficacious combinations (Figure 4). Initial screening identified meropenem and rifampicin as potential antibiotics of choice to use in combination with polymyxin B. However, rifampicin displays non-linear pharmacokinetics and has a number of toxicity issues [31], whereas the broad-spectrum bactericidal activity, wide safety margin and clinical familiarity of meropenem [32] led us to choose meropenem as the most promising candidate for clinical translatability. Subsequently, we systematically investigated the effect of polymyxin B/meropenem combinations on bacterial killing of planktonic bacteria, inhibition of biofilm formation, and membrane integrity analysis of the treatment-resistant population.

In the anti-biofilm effects and time-kill study, monotherapy with concentrations up to 16 mg/L for both polymyxin B and meropenem (and for carbapenem-resistant FADDI-PA060, meropenem 64 mg/L) were ineffective at 24 h, with substantial regrowth present at this time even when initial killing was present (i.e., with meropenem against FADDI-PA107 shown in Figure 6); biofilms were also present with all monotherapies (Figure 5). However, bacterial killing was significantly enhanced with combination therapy when each antibiotic was used at 16 mg/L. Indeed, against meropenem-susceptible FADDI-PA107, this combination resulted in complete eradication of planktonic bacteria and inhibition of biofilm formation (Figure 5). While a concentration of 16 mg/L is readily achievable in both plasma and epithelial lining fluid (ELF) for meropenem (discussed below), it is unachievable in both fluids for polymyxin B if administered intravenously at currently recommended doses [15,33,34]. Unfortunately, dose-limiting nephrotoxicity precludes intravenous polymyxin dose escalation to achieve higher concentrations [8]. However, inhaled polymyxin B and colistimethate (the latter the inactive prodrug of colistin [35]) are now often used as adjunct therapy with other antibacterial agents for the treatment of respiratory tract infections such as those occurring in patients with cystic fibrosis [29,36] and ventilator-associated pneumonia [37,38,39]. Administration via inhalation allows for a significant amount of drug to be delivered directly into the lungs while minimizing systemic exposure [34]. Indeed, polymyxin ELF concentrations ranging from 9.53 to 1137 mg/L have been reported in patients following pulmonary administration [15,33], and thus, a polymyxin B concentration of 16 mg/L is readily achievable in ELF following inhalation with standard doses.

For ß-lactam antibiotics, antibacterial activity has traditionally been correlated with the fraction of the dosing interval for which the unbound concentration remains above the MIC (*f*T_>MIC_) [40,41]. For meropenem, an *f*T_>MIC_ of ~40% is typically considered necessary for maximum bactericidal activity [40,41], although it has recently been suggested that a value of 100% *f*T_>4−5x MIC_ may be necessary to suppress resistance emergence [42]. ELF penetration of meropenem following intravenous administration of standard dosing regimens is variable, with meropenem ELF concentrations ranging from ~5 to 30 mg/L having been reported [43,44,45]. Thus, meropenem 16 mg/L is achievable in both plasma and ELF following intravenous administration [43,45].

Although rapid polymyxin bactericidal activity and maximal killing within the first hour against polymyxin-susceptible strains has been previously observed [46,47], the polymyxin B/meropenem combination did not increase the rate of early killing in these polymyxin-resistant strains (FADDI-PA060 polymyxin B MIC, 64 mg/L and FADDI-PA107 polymyxin B MIC, 32 mg/L). Based on their individual MICs (Table 1), it was no surprise that the meropenem-resistant FADDI-PA060 (meropenem MIC, 64 mg/L) showed minimal killing of planktonic bacteria and a failure to prevent biofilm formation with polymyxin and meropenem monotherapy even at 64 mg/L (Figure 5 and Figure 6). However, with meropenem monotherapy (16 mg/L), substantial initial killing at 4 h was observed against the meropenem-susceptible FADDI-PA107, followed by regrowth of planktonic bacteria and establishment of biofilm despite use of meropenem concentrations of ~4× MIC (Figure 5 and Figure 6). Sustained bacterial clearance for up to 24 h was only achieved with the simultaneous introduction of polymyxin B at 16 mg/L. Similar initial killing followed by regrowth of otherwise meropenem-susceptible clinical strains of *P. aeruginosa* has previously been reported in both planktonic and biofilm-embedded bacteria exposed to meropenem monotherapy with concentrations close to or even exceeding 100% *f*T_>MIC_ [48,49,50].

Formation of biofilm is a stress-induced response to environmental factors including antibiotic exposure [26,51]. In patients with indwelling medical devices and implants such as catheters, pacemakers, prosthetic devices and vascular grafts, the risk of biofilm-related infection is high. Indeed, ~60–70% of nosocomial infections are associated with implanted medical devices, with catheter-associated nosocomial infections accounting for ~36% of all healthcare-associated infections [52]. In patients with cystic fibrosis, the establishment of biofilms in lungs exacerbates non-specific immune responses that mediate chronic inflammation [53]. Once established, biofilms can tolerate antibiotic concentrations up to 10–1000-times those required to kill genetically equivalent planktonic bacteria which contributes to persisting infections with low eradication success [54]. Consequently, early intervention to inhibit biofilm formation and progression greatly improves the chances of successful bacterial eradication. Therefore, inhibition of biofilm formation through the proposed polymyxin B/meropenem combination is important and further investigations are warranted with a range of bacterial isolates in animal pharmacokinetic/pharmacodynamic models. Given polymyxins display an inoculum effect [47] and that our anti-biofilm evaluations were based on the initial stages of biofilm formation using a starting inoculum of ~10^6^ CFU/mL, the effect of the proposed combination on inhibiting their formation with higher starting inocula and/or eradicating mature biofilms should also be explored.

Inclusion of the meropenem-resistant strain FADDI-PA060 allowed investigation of the response of a doubly resistant isolate to the chosen combination. Combination therapy resulted in ~3 log_10_ CFU/mL greater killing of planktonic bacteria at 4 h than occurred with the most effective monotherapy, with growth at 24 h still ~4 log_10_ CFU/mL less than the control group (Figure 6). While bacterial killing of this isolate and inhibition of biofilm formation with the combination was not as effective as against FADDI-PA107, the initial ~3 log_10_ CFU/mL greater killing observed at 4 h with the combination may be important in patients, given such killing may reduce bacterial numbers sufficiently for clearance by the immune system [55]. Additionally, as will be discussed below, the viable cells present at this time contained significant membrane damage, which may make the cells more amenable to further treatments (Figure 7). As the time-kill model utilized lacks an immune system, especially granulocytes that work in concert with antibiotic treatment to kill bacteria [55], future animal studies and mechanism-based mathematical models incorporating an immune system effect are warranted to fully assess the utility of this combination against isolates resistant to both antibiotics.

It is likely the different mechanisms of action and resistance of each antibiotic contributed to the enhanced bacterial killing observed with the combination. While the precise mechanism(s) by which polymyxins kill bacteria is uncertain, the initial target of polymyxins against Gram-negative bacteria is lipopolysaccharide (LPS) in the outer membrane, and the electrostatic attraction between cationic amine moieties on the polymyxin and anionic phosphate and carboxylate moieties on LPS is critical for activity [56]. Binding results in displacement of the native divalent cations and considerable outer membrane disorganization, and ultimately cell death. Membrane disruption increases membrane permeability not only to polymyxin itself but also other compounds [57]. Most identified polymyxin resistance mechanisms involve changes in LPS that negate this initial interaction [56]. While both isolates in the present study were polymyxin-resistant, membrane integrity studies involving FADDI-PA060 nevertheless showed that polymyxin B was still able to cause considerable membrane disruption (evidenced by membrane depolarization and increased membrane permeability) in most bacterial cells, with a subpopulation of cells markedly affected (Figure 7A,B). Changes associated with membrane remodeling combined with disruption of the outer membrane by polymyxin B likely improved access of meropenem to their target proteins, namely the penicillin-binding proteins located on the outside of the cytoplasmic membrane [32]. Increased intracellular meropenem concentrations by polymyxin B treatment (i.e., bioavailability synergy [58]) would help to negate meropenem resistance which occurs primarily due to enzymatic inactivation via carbapenemases and reduced entry into the cell via a reduction in the expression of the outer membrane porin OprD [59].

In addition to increased bioavailability of meropenem, we have previously demonstrated in colistin-susceptible *Acinetobacter baumannii* that colistin monotherapy causes disruption to the bacterial cell wall in addition to bacterial membranes [60]. Furthermore, doripenem alone disrupted peptidoglycan biosynthesis, whereas a combination of colistin and doripenem induced significant time-dependent changes to more key metabolic pathways relative to either monotherapy. Specifically, cell wall biosynthesis was down-regulated initially by colistin (across the first hour of treatment) and later by doripenem (4 h). Thus, it is possible that enhanced bacterial killing in the present study may additionally result from disruption of the cell wall by both polymyxin B and meropenem. The use of such combinations also reduces the chances of the emergence of resistant mutants as separate and independent mutations are required for resistance development [61]. Interestingly, although regrowth of FADDI-PA060 nevertheless occurred following substantial initial killing, the cells at 4 h exhibited significant membrane depolarization and increased permeabilization thus, indicating loss of overall membrane integrity (Figure 7A,C). It is unclear if this damage is sufficient to prevent additional membrane restructuring that further reduces susceptibility to these antibiotics. The effect such damage has on these cells’ response to further treatment and/or immune effects should be investigated.

In summary, we identified polymyxin/meropenem as a promising combination against biofilm-producing, polymyxin-resistant MDR *P. aeruginosa* and systematically examined bacterial killing of planktonic bacteria, inhibition of biofilm formation and membrane integrity. Further evaluations in animal infection models will facilitate the translation into the clinic.

## 4. Materials and Methods

### 4.1. Antibiotics, Bacterial Strains, Media and Susceptibility Testing

Stock solutions of polymyxin B sulfate (Batch number 20120204; Beta Pharma Co Ltd., Shanghai, China), colistin sulfate (Batch number 20120507; Beta Pharma Co Ltd., Shanghai, China), azithromycin dihydrate (Batch number PHR1088-1G; Sigma-Aldrich, Castle Hill, NSW, Australia), cefepime (Batch number DJP-100307; Cgene Tech, Suzhou, China), ciprofloxacin (Batch number 065M4176V; Sigma-Aldrich, Castle Hill, NSW, Australia), meropenem trihydrate (Batch number MAUS1029; Fresenius Kabi, North Ryde Bc, NSW, Australia) and rifampicin (Batch number SLBK5059V; Sigma-Aldrich, Castle Hill, NSW, Australia) were used. Polymyxin B, colistin and meropenem stocks were prepared using Milli-Q water (Millipore Australia, North Ryde, New South Wales, Australia) immediately prior to each experiment and sterilized by filtration with a 0.22-µm Millex-GV PVDF filter (Millipore, Bedford, MA, USA). Meropenem stock was sonicated for 2 min prior to filter sterilization. Azithromycin, cefepime and rifampicin were dissolved in 25% (*v*/*v*) dimethyl sulfoxide (Sigma-Aldrich, Castle Hill, NSW, Australia) and 75% (*v*/*v*) Milli-Q water, followed by filter sterilization through a 0.22-µm Millex-FG PTFE filter (Millipore, Bedford, MA, USA). Ciprofloxacin was dissolved in 0.1 M HCl (Supelco, Bellefonte, PA, USA) and filtered through a 0.22-µm Millex-GV PVDF filter (Millipore, Bedford, MA, USA).

Two MDR strains of *P. aeruginosa* collected from patients with cystic fibrosis (FADDI-PA060 and FADDI-PA107) were examined. MDR was defined as non-susceptibility to at least one agent in at least three of the following antimicrobial classes: aminoglycosides, antipseudomonal carbapenems, antipseudomonal cephalosporins, antipseudomonal fluoroquinolones, antipseudomonal penicillins plus ß-lactamase inhibitors, monobactams, phosphonic acids and polymyxins [22]. *P. aeruginosa* ATCC 27853 was used as the quality control strain. Strains were stored in tryptone soy broth (Oxoid, Basingstoke, Hampshire, England, UK) with 20% (*v*/*v*) glycerol at −80 °C. Prior to each experiment, strains were subcultured onto nutrient agar (School of Biomedical Sciences Media Unit, Monash University, Australia) and incubated at 37 °C for 24 to 48 h. Cation-adjusted Mueller-Hinton Broth (CAMHB; Ca^2+^ at 23 mg/L, Mg^2+^ at 12.2 mg/L; Oxoid, Hampshire, England, UK) was employed for all microbiological experiments.

### 4.2. Genome Sequencing, Assembly and Annotation

Bacterial log-phase cultures were used to extract genomic DNA with a DNeasy^®^ Blood and Tissue Kit (QIAGEN, Hilden, Germany) as per the manufacturer’s instructions. DNA quality and quantity were assessed using electrophoresis and Qubit (Life Technologies, Carlsbad, CA, USA) before Illumina HiSeq sequencing (paired-end 150 bp, Genewiz, Suzhou, China) [62]. *De novo* assembly was conducted using SPAdes [62,63] followed by annotation with NCBI Prokaryotic Genome Annotation Pipeline (PGAP). The genome sequences and annotations are available under accession number PRJNA664899. The sequence types were determined using PubMLST [64]. Antibiotic resistance genes were predicted using the Resistance Gene Identifier (RGI) 3.2.1 tool of the Comprehensive Antimicrobial Resistance Database (CARD) [18]. Putative virulence factors of FADDI-PA060 and FADDI-PA107 were predicted by blasting the whole genome against the Virulence Factor Database (VFDB) [65].

### 4.3. Population Analysis Profiles (PAPs)

Minimum inhibitory concentrations (MICs) prior to drug exposure were determined in duplicate [66]. Susceptibility and resistance were determined according to the European Committee of Antimicrobial Susceptibility Testing (EUCAST) guidelines [23].The possible existence of polymyxin-resistant subpopulations at baseline was assessed using population analysis profiles (PAPs; inoculum, ~10^8^ CFU/mL). A 50 µL sample of an appropriately diluted overnight culture was manually plated on to Mueller–Hinton agar (School of Biomedical Sciences Media Unit, Monash University, Australia) containing polymyxin B at concentrations of 0, 0.5, 2, 4 or 16 mg/L. Plates were incubated in an incubator at 37 °C for 48 h and colonies counted using an automated colony counter (ProtoCOL3, Synbiosis, Cambridge, United Kingdom). The limit of detection was 20 CFU/mL (=1.3 log_10_ CFU/mL, equivalent to 1 colony per plate).

### 4.4. Checkerboard Studies

An initial screen for synergy was conducted with polymyxin B in combination with rifampicin, meropenem, azithromycin and cefepime using the checkerboard microbroth dilution method [67]. These antibiotics were considered representative antibacterial agents from a variety of antibacterial classes. Following 24 h of incubation at 37 °C, absorbance was read using a multimode plate reader at λ = 600 nm. Synergistic combinations (i.e., fractional inhibitory concentration index of ≤0.5) were identified in order to decide which combination and concentrations would be employed in subsequent time-kill and biofilm studies.

### 4.5. Biofilm-Formation and Time-Kill Study

Based on the checkerboard study results, bacterial killing and the effect on biofilm formation of polymyxin B and meropenem were subsequently examined. An initial conditioning phase was undertaken using a previously described static biofilm model to assess the in vitro biofilm-forming capacity of each isolate [68]. A mid-log phase culture of each strain (Denville^®^ CO8000 Cell Density Meter reading 0.5, equivalent to ~10^8^ CFU/mL) was diluted 1:100 with CAMHB to achieve a starting inoculum of ~10^6^ CFU/mL. Subsequently, 100 µL aliquots were inoculated into a non-pyrogenic flat-bottomed microtitration plate (Thermo Scientific, Brisbane, Australia) containing 100 µL of CAMHB. In order to determine the optimum conditioning phase time, one plate was initially incubated at 37 °C for 24 h and a second plate for 48 h. Following incubation, plates were washed three times with 200 µL phosphate-buffered saline (PBS, pH 7.4) to remove planktonic cells and stained with 220 µL of 0.1% (*w*/*v*) crystal violet solution. The stained biofilm was solubilized with 200 µL of 30% (*v*/*v*) acetic acid. Absorbance (OD) was measured at λ = 550 nm using a multimode plate reader (Infinite M200, Tecan, Mennendorf, Switzerland) [68,69]. Results from 6 technical replicates were used to categorize each strain as a biofilm producer or non-biofilm producer using the method of Stepanovic et al. [69]. An OD value of >2 × OD of the negative control indicated biofilm production [69]. Following confirmation of biofilm formation, the inhibitory effect of antibiotic therapy on biofilm formation were examined. Polymyxin B 16 mg/L and meropenem 16 mg/L were examined as monotherapy and in combination with staining undertaken following 24 h of treatment at 37 °C as described above. Changes in optical density (OD) corresponding to the change in biofilm formation were determined, and results were calculated using six replicates from two independent experiments.

To examine changes in bacterial killing and biofilm formation, time-kill studies were undertaken as described previously with minor modifications where necessary to allow for biofilm formation [70]. Briefly, a mid-log phase inoculum of each bacterial strain was diluted 1:100 in a sterile 50-mL polypropylene tube (Greiner Bio One, Kremsmunster, Austria) with CAMHB (final volume, 20 mL) to establish a starting inoculum of ~10^6^ CFU/mL. A sterile Teflon coupon (Biosurface Technologies, Bozeman, MT, USA) was added to each 50 mL tube at the beginning of the experiment (t = 0 h) for biofilm measurement. Tubes were placed in a shaking water bath (shaking speed 200 rpm) [71], and the experiment was conducted over 24 h at 37 °C. For monotherapy against FADDI-PA060, polymyxin B was added at 4, 8 and 16 mg/L and meropenem at 16 and 64 mg/L. For FADDI-PA107, the concentrations used were 1, 2, 4 and 16 mg/L for polymyxin B and 1, 2, 4 and 16 mg/L for meropenem. Five combinations (polymyxin B 4 mg/L plus meropenem at 4, 8, or 16 mg/L, polymyxin B 8 mg/L plus meropenem 8 mg/L, and polymyxin B at 16 mg/L plus meropenem at 16 mg/L) were examined against FADDI-PA060. Four combinations (polymyxin B 1 mg/L plus meropenem 1 mg/L, polymyxin B 2 mg/L plus meropenem 2 mg/L, polymyxin B 4 mg/L plus meropenem 4 mg/L and polymyxin B 16 mg/L plus meropenem 16 mg/L) were examined against FADDI-PA107. Following 24 h of treatment, 0.5 mL of sample was removed for enumeration of planktonic bacteria, centrifuged at 10,000× *g* for 10 min and resuspended in sterile saline to minimize antibiotic carryover. For enumeration of biofilm-embedded bacteria, coupons were removed and biofilm-embedded cells recovered [72]. For both planktonic and biofilm-embedded cells, 50 µL of undiluted or appropriately diluted sample was spirally plated onto nutrient agar plates before incubation at 37 °C for 48 h. Colonies were counted as per PAPs and each regimen was performed in triplicate. The most effective combination was examined via an additional time-kill study on planktonic growth across 24 h (1, 2, 4 and 24 h).

### 4.6. Membrane Integrity Assay

Bacterial membrane integrity at 4 h post-treatment was determined using flow-cytometry [25]. Samples were assessed using blue (BL) and violet (VL) laser detection channels of the ACEA NovoCyte^®^ high-performance benchtop flow cytometer (ACEA Biosciences, Santa Clara, CA, USA). The voltage-sensitive fluorophore Bis-(1,3-Dibutylbarbituric Acid) Trimethine Oxonol (DiBAC) (Sigma-Aldrich, Castle Hill, NSW, Australia) was used as an indicator of bacterial membrane depolarization [24]. Propidium iodide (PI) (Sigma-Aldrich, Castle Hill, NSW, Australia), a live cell impermeant fluorophore, tagged bacterial cells with damaged cell membranes [24,25,73]. Both DiBAC (Ex/Em 488/660–690 nm) and PI (Ex/Em 488/660–690 nm) were detected in the BL-4 channel. A threshold forward-scatter (FSC-H) and side-scatter height (SSC-H) of >1000 units and events/s <1000 were enforced. A maximum acquisition of 20,000 events was set in the study [73]. Six hundred microliters of the appropriately diluted samples were separately stained with 1.67 mg/L DiBAC and 8.33 mg/L PI, respectively. Samples were incubated at 37 °C for 1–2 min and vortexed prior to analysis. The mean fluorescence intensities (FI) of DiBAC and PI correspond to the relative stain binding capacity of each cell [25]; FI data are presented as fold-changes relative to non-treated controls.

Flow cytometry data were normalized by the mean, log transformed and analyzed using the NovoExpress^®^ software (V 2.1 ACEA Biosciences, Santa Clara, CA, USA).

### 4.7. Data Processing and Statistical Analysis

All Experimental raw data were analyzed by one-way ANOVA using GraphPad Prism 8 (GraphPad Software Inc., San Diego, CA, USA) (*n* = 3).

## Figures and Tables

**Table 1:** Minimum inhibitory concentrations (MICs).

Antibacterial Agent	FADDI-PA060		FADDI-PA107	
	MIC (mg/L)	Interpretation	MIC (mg/L)	Interpretation
Colistin	>128	R	32	R
Polymyxin B *	64	R	32	R
Rifampicin	8	NA	16	NA
Meropenem	64	R	4	S
Azithromycin	128	NA	>128	NA
Cefepime	>128	R	>128	R
Ciprofloxacin	32	R	<0.125	S

* The EUCAST breakpoints for colistin were applied to polymyxin B [23]. Susceptibility (S) and resistance (R) were defined as MICs ≤ 2 and >2 mg/L for colistin and polymyxin B, and ≤2 and >8 mg/L for meropenem. Intermediacy and resistance were defined as MICs ≤ 8 and >8 mg/L for cefepime and ≤0.5 and >0.5 mg/L for ciprofloxacin [23]. Breakpoints for azithromycin and rifampicin against Gram-negative bacteria are not available (NA).

## Data Availability

Data are contained within the article or supplementary material.

## Supplementary Materials

The following are available online at https://www.mdpi.com/article/10.3390/antibiotics10040405/s1. Figure S1: Visual representation of crystal violet staining patterns corresponding to biofilm bacterial growth following 24 h of either no treatment (growth controls) or treatment with polymyxin B and/or meropenem (each at 16 mg/L) in the static biofilm model (*n* = 6).
