# Supplementary material for: Clinically Relevant Concentrations of Polymyxin B and Meropenem Synergistically Kill Multidrug-Resistant Pseudomonas aeruginosa and Minimize Biofilm Formation
Source: Antibiotics (Basel). 2021 Apr 8;10(4):405. doi: 10.3390/antibiotics10040405 (PMC8069709; doi:10.3390/antibiotics10040405)
Supplement: Supplementary file 1 [file antibiotics-10-00405-s001.pdf]

Article

Clinically Relevant Concentrations of Polymyxin B and Meropenem Synergistically Kill Multidrug-Resistant *Pseudomonas aeruginosa* and Minimize Biofilm Formation

Hasini Wickremasinghe [1,*], Heidi H. Yu [1], Mohammad A. K. Azad [1], Jinxin Zhao [1], Phillip J. Bergen [1], Tony Velkov [2], Qi Tony Zhou [3], Yan Zhu [1] and Jian Li [1]

[1] Infection and Immunity Program, Department of Microbiology, Biomedicine Discovery Institute, Monash University, Clayton, VIC 3800, Australia; heidi.yu@monash.edu (H.H.Y.); mohammad.azad@monash.edu (M.A.K.A.); jinxin.zhao@monash.edu (J.Z.); phillip.bergen@monash.edu (P.J.B.); yan.zhu@monash.edu (Y.Z.); jian.li@monash.edu (J.L.)

[2] Department of Pharmacology and Therapeutics, School of Biomedical Sciences, Faculty of Medicine, Dentistry and Health Sciences, The University of Melbourne, Parkville, VIC 3053, Australia; tony.velkov@unimelb.edu.au

[3] Department of Industrial and Physical Pharmacy, Purdue University, West Lafayette, IN 1047907, USA; tonyzhou@purdue.edu

* Correspondence: hasini.wickremasinghe@monash.edu (H.W.); Tel.: +61-3-9903-9251; Fax: +61-3-9905-6540

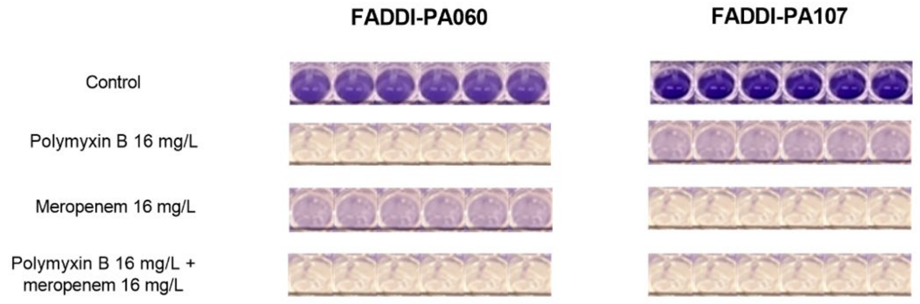

Figure S1. Visual representation of crystal violet staining patterns corresponding to biofilm bacterial growth following 24 h of either no treatment (growth controls) or treatment with polymyxin B and/or meropenem (each at 16 mg/L) in the static biofilm model (n = 6).
